# Association between Anti-DENV IgM Serum Prevalence and CD11b Expression by Classical Monocytes in Obesity

**DOI:** 10.3390/v15010234

**Published:** 2023-01-14

**Authors:** Karine Beatriz Costa, Bruna Caroline Chaves Garcia, Marina Luiza Baêta Costa, Yara Gomes Pena, Eduardo Augusto Barbosa Figueiredo, Marcelo Henrique Fernandes Ottoni, Juliane Duarte Santos, Vinícius de Oliveira Ottone, Danilo Bretas de Oliveira, Etel Rocha-Vieira

**Affiliations:** 1 Programa Multicêntrico de Pós-Graduação em Ciências Fisiológicas, Universidade Federal dos Vales do Jequitinonha e Mucuri, Diamantina 39100-000, MG, Brazil; 2 Departamento de Medicina, Universidade Federal de Juiz de Fora, Governador Valadares 35010-180, MG, Brazil; 3 Laboratory of Exercise Biology and Immunometabolism, Centro Integrado de Pós-Graduação e Pesquisa em Saúde, Universidade Federal dos Vales do Jequitinonha e Mucuri, Diamantina 39100-000, MG, Brazil; 4 Programa de Pós-Graduação em Ciências da Saúde, Universidade Federal dos Vales do Jequitinonha e Mucuri, Diamantina 39100-000, MG, Brazil

**Keywords:** body fat index, inapparent dengue, monocyte subsets

## Abstract

Dengue and obesity are currently highly prevalent conditions worldwide and the association between these two conditions may result in greater risk for DENV infection and disease severity. In this study the association between obesity and recent, inapparent dengue was investigated. Serum DENV IgM and NS1 were evaluated in 49 adult volunteers (15 lean and 34 individuals with obesity, according to body mass index), between September 2017 and June 2018. Adiposity, endocrine, metabolic, and immune data of the participants were also obtained. None of the study participants tested positive for the DENV NS1 antigen. DENV IgM was detected in 33.3% of the lean individuals, and in 44.1% of those with obesity; the presence of DENV IgM was not associated with body mass index (OR = 1.32, 95% CI = 0.59–2.98, *p* = 0.48). However, body fat index was higher in obese individuals who had recent inapparent dengue (14.7 ± 3.1 versus 12.7 ± 2.1 kg/m^2^, *p* = 0.04), as was the expression of CD11b by classical (CD14^++^CD16^−^) monocytes (1103.0 ± 311.3 versus 720.3 ± 281.1 mean fluoresce intensity). Our findings suggest an association between adiposity and recent inapparent dengue and the involvement of classical monocytes in this association.

## 1. Introduction

Dengue is the tropical neglected, mosquito-borne viral infection with the highest incidence worldwide [1], caused by four different serotypes of dengue virus (DENV) and single-stranded, positive-sense RNA viruses (DENV-1 to -4) [2]. The disease is endemic in more than 100 countries in the World Health Organization (WHO) regions of Africa, the Americas, the eastern Mediterranean, Southeast Asia, and the Western Pacific and its global incidence increased from 505,430 cases in 2000 to more than 5 million cases in 2019 [3].

Most human infections (>70%) by DENV are inapparent or asymptomatic, with no clinical manifestations or mild symptoms that do not result in sufficient discomfort for clinical consultation [2,4,5]. Despite having no implication on clinical management, inapparent infections can impact disease epidemiology and dynamics. Otherwise, apparent infection is often a self-limiting febrile illness, with a wide range of clinical manifestations including headache, nausea, vomiting, rash, and joint and muscle pain [6,7]. However, a small proportion of dengue cases progress into a life-threatening condition, with hemorrhagic manifestations, multi-organ impairment, plasma leakage, and shock syndrome, known as severe dengue [6,7].

Multiple viral and host factors determine the development of severe forms of dengue, which include, amongst others, secondary infections and virus genetics, patient age and gender and the presence of chronic diseases such as diabetes, hypertension, coronary artery disease, and chronic renal failure [8,9,10,11]. More recently the association between dengue severity and obesity has been proposed [12,13,14].

According to the WHO, overweight and obesity has tripled since 1975 and it is estimated that almost 60% of the world population will be overweight or have obesity by 2030 [15,16,17]. The increase of overweight and obesity in the regions with a high risk of dengue infection is noteworthy: the Southeast Asian, Western Pacific, and African regions were those with the lowest prevalence of obesity in 1980 (between 0.4–2.0%), but in 2015 obesity prevalence reached 6.2% in the Southeast Asia, 4.9% in the Western Pacific, and 12.7% in Africa [15]. The trends in overweight and obesity also increased in the Americas and the eastern Mediterranean regions during the past 35 years as well [15].

The expansion of the adipose organ in obesity is associated with the settlement of low chronic inflammation, also named meta-inflammation, in this and other organs responsible for the systemic energy homeostasis control [18,19]. Meta-inflammation not only disturbs the metabolic and endocrine role of adipose, skeletal muscle, and hepatic organs, but can also impact the immune function [20]. Alterations in the distribution, activation, and the secretory function of the monocyte subsets (the DENV target cells) are consistently observed in individuals with obesity [21,22,23,24]. It is proposed that, amongst other factors, the endocrine disturbances associated with obesity, such as insulin resistance and hyperleptinemia, can be linked to the immune imbalance associated with this condition [25,26]. Moreover, glucose and fatty acid excess can also contribute to immune dysfunction in obesity [27,28]. The immune imbalance associated with obesity can lead to increased susceptibility to infectious diseases [20,29,30].

In this study, we investigated whether obesity biomarkers can be related to previous, recent, inapparent DENV infection. To this, lean and individuals with obesity with a recent history of inapparent dengue (DENV IgM), were compared for metabolic, endocrine, adiposity and immune parameters.

## 2. Materials and Methods

This was a cross-sectional, case-control study that investigated the association between obesity and recent, inapparent DENV infection. Inapparent dengue was confirmed by the presence of anti-DENV IgM, evaluated in serum samples of lean and individuals with obesity (convenience sample), and collected during the peak period of dengue in Brazil, between September 2017 and June 2018. The study was approved by the Universidade Federal dos Vales do Jequitinhonha and Mucuri institutional review board (CAAE 42613321.3.0000.5108). All procedures were in accordance with the ethical standards of the Helsinki Declaration and participants provided written informed consent.

Fifty individuals of both sexes, all non-smokers, between the ages of 18 and 42 years-old participated in this study. Volunteers were recruited from the local community based on the following criteria: not being engaged in exercise or weight-loss program for at least six months prior to the study (self-reported); having a stable body weight (±2 kg) for at least three months prior to the study (self-reported); no use of anti-inflammatory, hypoglycemic, or any other medication known for affecting metabolism; and the absence of diabetes, hypertension, and self-reported acute or chronic diseases. Individuals who had undergone immunization in the 30 days prior to the study, who had experienced any symptomatic infectious condition in the previous 15 days, or who presented any symptoms of infectious disease in the moment of the study, were also not considered. Those with altered glucose metabolism (fasting plasma glucose > 100 mg/dL or end point 2-h oral glucose tolerance test plasma glucose > 140 mg/dL) were also not admitted to the study. None of the participants reported previous and recent occurrence of dengue diagnosis and no record of the participants in the National Health System for dengue surveillance was found.

Participants were stratified according to their body mass index (BMI) into “lean” or “with obesity” groups. BMI cutoff values for lean and obesity were <25 kg/m^2^ and ≥30 kg/m^2^, respectively. Participants with obesity were further stratified according to their insulin status into “insulin sensitive” and “insulin resistance” groups. Insulin resistance (HOMA1-IR) was estimated from fasting glucose and insulin [31]. The cutoff value to classify insulin resistance was HOMA1-IR ≥ 2.71 [32].

The qualitative detection of anti-DENV IgM was performed by ELISA, using the Panbio^®^ Dengue IgM Capture ELISA kit (Queensland, Australia). Results are reported as negative, positive, or equivocal according to the cut-off value determined from dengue endemic populations from Southeast Asia and South America. Samples with equivocal results were tested twice and excluded from the study if the equivocal result remained. The test has a primary serological sensitivity of 94.7% (CI 95%: 85.4–98.9%), negative serological specificity of 100% (CI 95%: 95.7–100%), and secondary serological sensitivity of 55.7% (95% CI: 46.6–64.7%). Samples were also tested for NS1 viral antigen, using an immunochromatographic lateral flow test (Quibasa Química Básica Ltd.a, Brazil). The result is qualitatively interpreted as reactive (when NS1 antigen is present) or non-reactive (when NS1 antigen is absent). The test has a clinical sensibility of 94.4%, and a specificity of 99.0%.

Body mass index (BMI) was calculated from height and body weight values. Dual X-ray absorptiometry (DXA, Lunar, DPX, Madison, Wisconsin, USA) was used to assess body fat percentage (BFP), body fat mass (BFM), and visceral fat mass (VFM). Waist circumference was also measured (midway between the lower rib margin and the iliac crest). Body fat index (BFI) was calculated from the ratio between adipose mass and the square of height. Based on BFI, individuals with obesity were categorized in obesity grades I to III [33].

After a 12-h fasting period, serum levels of insulin, leptin, glucose, total cholesterol, and its fractions (LDL, HDL and VLDL) were measured according to the Clinical and Laboratory Standards Institute (CLSI) guidelines. Measurements were conducted at a local Clinical Laboratory, using standardized chemiluminescence (insulin) and enzymatic/colorimetric methods (glucose and lipids). Leptin levels were determined by ELISA (Human Leptin Duoset ELISA, R&D Systems), according to the manufacturer instructions. Pancreatic beta cell secretory function (HOMA-β) was estimated from blood levels of fasting glucose and insulin [31]. The volunteers were also submitted to the oral glucose tolerance test (OGTT), in which blood glucose concentration was determined at 0, 30, 60, 90, and 120 min. The 2-h blood glucose area under the curve (AUC) was calculated by the trapezoidal method, using the GraphPad Prism (version 7.00 for Mac OS X, GraphPad Software, San Diego, CA, USA). Total and differential blood leukocyte counts were also conducted, using an automatic cell counter (Auto Hematology Analyzer BCE 5380, Mindray, Shenzhen, China).

Flow cytometry was used to determine the percentage of monocyte subsets using 50 μL of whole blood, collected after 12-h overnight fast, as previously described [21]. The following fluorochrome-conjugated antibodies were used: CD14-APC (18D11), CD16-APC-Cy7 (3G8), HLA-DR-FITC (G46-6), and CD11b-PE (ICRF44), as well as rat anti-mouse IgG1 (A85-1) and IgG2a + b (X57). The antibodies were all branded BD Pharmingen. The percentage of classical (CD14^++^CD16^−^), intermediate (CD14^++^CD16^+^) and non-classical (CD14^+^CD16^++^) monocytes was determined from the total monocyte population, as proposed by Abeles et al. [34]. The expression of HLA-DR and CD11b by the three monocyte subsets was also evaluated (mean fluorescence intensity—MFI). The FACSCanto II flow cytometer (Becton Dickinson, San Jose, CA, USA) was used. Fifty thousand events in the monocyte region were acquired, and data were analyzed using the Flow-Jo 10.0.8r1 (FlowJo, LCC 2006–2015, Ashland, OR, USA) software. Non-stained, isotype and fluorescence minus one (FMO) controls were used to establish gate and/or quadrant positioning.

Categorical data were expressed as absolute and relative frequencies and the IBM SPSS (version 22.0, IBM Corp., Armonk, NY, USA) was used for statistical analysis. Data were compared using Person’s chi-square test. The odds ratio (OR) was used to determine the magnitude of the relation between the variables (risk estimate), with a confidence interval (CI) of 95%. The category of reference was determined respecting the hierarchy between possible factors associated with the outcome. Associations were considered significant when the CI of OR did not include the value 1.00 (*p* < 0.05). Statistical power was determined using the GraphPad StatMate 2.00 software (GraphPad Software, San Diego, CA, USA). Continuous data were expressed as mean ± standard deviation, and GraphPad Prism (version 7.00 for Mac OS X) was used for statistical analysis. The Shapiro–Wilk test evaluated data normality. Given that the dependent variables were normally distributed, the one-way analysis of variance (ANOVA) compared data amongst lean and obesity groups, according to anti-DENV IgM status. Significant difference between means was determined by the Šídák multiple comparison test corrected for multiple comparisons using statistical hypothesis testing. *p* ≤ 0.05 was considered statistically significant.

## 3. Results

None of the individuals included in this study were reactive for the NS1 antigen, confirming no active DENV infection. Of the 50 individuals investigated, 29 were negative for DENV IgM and 20 were positive, suggesting recent inapparent dengue. One participant had an equivocal result after two independent tests and was excluded from the study. Of the 49 individuals with conclusive results for DENV IgM evaluation, based on BMI categorization, fifteen (30.6%) were lean and 34 (69.4%) had obesity. DENV IgM was detected in 5 (33.3%) of the 15 lean individuals, and amongst the individuals with obesity, 15 (44.1%) were positive for this antibody. According to the BMI categorization, the presence of DENV IgM was not associated with obesity (OR: 1.32, 95% CI: 0.59–2.98, *p* = 0.48). Thus, considering the BMI, it cannot be said that recent, inapparent dengue is associated with obesity.

Because obesity can be a risk factor for infectious diseases and its complications, the association between adiposity, metabolic and endocrine data, and inapparent dengue was evaluated. As shown in Table 1, body mass, BMI, waist circumference, body fat mass and percentage, visceral fat mass, and lean tissue mass did not influence the comparison of individuals in terms of the presence or absence of DENV IgM. However, BFI amongst individuals with obesity was higher in those who were positive for DENV IgM, compared with the DENV IgM negative individuals (*p* = 0.04), suggesting an association between adiposity and inapparent DENV infection. Individuals with obesity were categorized as obese grade I, II, and III, according to the BFI. Amongst those with recent inapparent dengue (DENV IgM positive), the percentage of obesity grade I, II, and III was of 57.1%, 21.9%, and 20.0%, respectively. While between DENV IgM negative participants, 77.8% had obesity grade I and 22.2% were categorized as grade II, with no participant presenting obesity grade III. However, no association between recent inapparent dengue and obesity grade was observed (OR: 1.69, IC 95%: 0.921–2.750, *p* = 0.07—obesity grades II and III versus grade I).

Moreover, no difference in endocrine and metabolic parameters (fasting glucose and insulin, leptin, HOMA-IR, HOMA-β, total cholesterol, HDL, LDL, and triglycerides) were observed comparing individuals according to the DENV IgM and obesity status (Table 2). However, VLDL levels were reduced in the obesity group of individuals with recent inapparent dengue (*p* = 0.04). When individuals with obesity were categorized according to the insulin sensitivity status, 10 of the 19 insulin-resistant individuals (52.6%) were seropositive for DENV IgM, while only 5 of the 15 insulin-sensitive individuals (33.3%) presented DENV IgM. The presence of DENV IgM was independently associated with insulin resistance (*p* = 0.26, 20% power), with a 1.58-fold higher chance of insulin-resistant individuals being positive for DENV IgM (OR: 1.58, 95% CI: 0.686–3.632).

**Figure 1:**
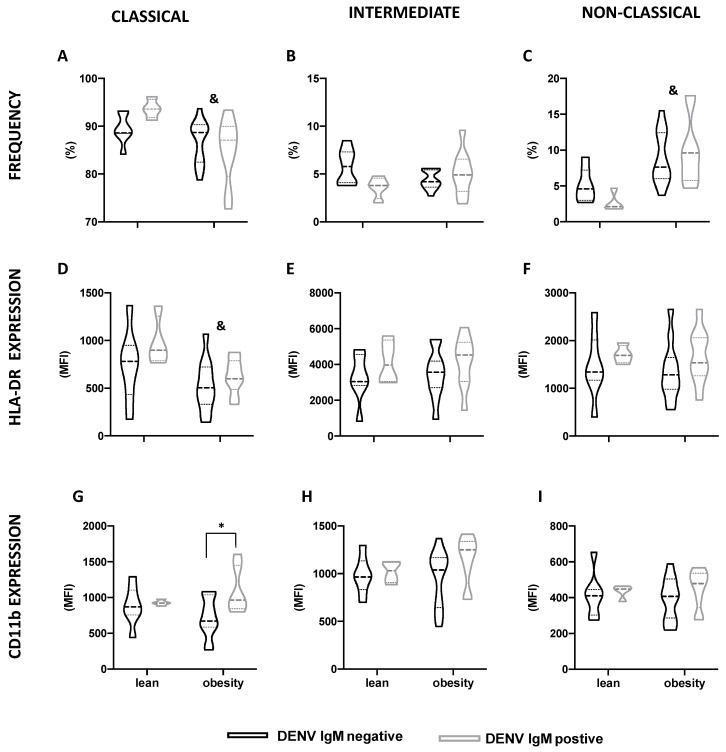
Monocyte subsets profile according to BMI and DENV IgM status. Classical (**A**,**D**,**G**), intermediate (**B**,**E**,**H**), and non-classical (**C**,**F**,**I**) monocytes distribution (**A**–**C**), HLA-DR (**D**–**F**) and CD11b expression (**G**–**I**) in lean individuals and individuals with obesity, according to DENV IgM status. Data are expressed as median (dashed line) and the first and third quartiles (dotted lines). ^&^ *p* < 0.05, lean versus obesity, irrespective of the DENV IgM status, two-way Anova. * *p* < 0.05, DENV IgM negative versus DENV IgM positive, two-way Anova, Šídák’s multiple comparison test.

The global and differential blood leukocyte counts, according to the anti-DENV IgM status, were not different (Table 3). Despite the lower percentage of classical monocytes, and the higher percentage of non-classical monocytes in individuals with obesity compared to lean individuals, no difference in the monocyte subsets, according to anti-DENV IgM status, was observed (Figure 1A–C). The same was found for the HLA-DR expression by different monocyte subsets (Figure 1D–F). However, amongst individuals with obesity, the CD11b expression by classical monocytes was greater (*p* = 0.008) in those who had previously inapparent dengue, compared to anti-DENV IgM negative ones (Figure 1G).

## 4. Discussion

In this study we investigated whether obesity biomarkers can be associated to recent inapparent DENV infection, using the presence of serum DENV IgM as the indicator of recent dengue. No association between obesity and metabolic and endocrine obesity markers and inapparent dengue were observed. However, amongst individuals with obesity, body fat excess and the expression of CD11b by classical monocytes was higher in those who had recent, inapparent dengue.

Independent of BMI status, a high prevalence of DENV IgM (40.8%, considering both lean and obesity) was observed is this study. Although this finding is limited by the nature of the study (convenience sample), it can have two significant implications. First, inapparent infections are of epidemiological significance because asymptomatic individuals can be infectious to mosquitoes and, at a given level of viremia, asymptomatic DENV-infected people are more infectious to mosquitoes [35]. In Brazil, symptomatic dengue incidence in 2017 and 2018 was one of the lowest [36], and the reduction in dengue incidence in this period was attributed, at least in part, to the population immunity established during the preceding years, when dengue incidence was very high [37].

In recent years the association between obesity and dengue severity has been proposed. Amongst dengue hospitalized patients, epistaxis and hepatitis, for examples, as well as thrombocytopenia, were more prevalent in those with obesity, although the association between obesity and dengue severity or dengue mortality had not been observed [12,14]. Only Chiu et al. [13] reported a 17% higher chance of dengue severity in adult patients with obesity.

As in many other reports, in this study, obesity was defined according to BMI and no association between obesity and dengue was found using this criterion for obesity definition. However, when DENV IgM positive and negative individuals with obesity were compared by BFI, those with a BMI ≥ 30 kg/m^2^ who had a recent, inapparent dengue also presented a higher BFI, suggesting an association between body adiposity and DENV infection. Given that BMI provides a measure of excess weight, not excess fat, and that it does not account for gender or ethnicity, our data indicate that more sensible or accurate measures of body fat excess should be preferred in studies that seek to establish the association between obesity and other clinical conditions.

Moreover, in the present study, we report for the first time in individuals with obesity who had recent inapparent DENV infection (albeit not in lean ones), an association between CD11b expression by classical monocytes and dengue. Monocytes are key contributors to both dengue pathophysiology and disease control [38,39] and major transcriptional changes are observed in monocyte subsets in DENV-infected patients [40]. Augmented CD11b expression by non-classical monocytes during active, symptomatic dengue was previously reported, and it was correlated with the magnitude of the inflammatory response triggered by the disease [41]. A role for CD11b in monocyte infection by DENV has also been proposed [42]. In mice, DENV infection leads to the recruitment of Ly6C^high^ monocytes—the functional correspondent of human classical monocytes— to the dermis, where they differentiate into Ly6C^high^CD11b^+^ monocyte-derived dendritic cells and become the major target for virus replication [43]. We cannot say whether monocyte CD11b expression was up-regulated in response to dengue in the individuals with obesity and if so, why this did not occur in lean individuals. We also cannot say whether the individuals with obesity had elevated CD11b expression by monocytes before infection. Despite this limitation, as monocytes are involved in both obesity and dengue pathophysiology, these cells could be the mechanistic link between obesity and dengue severity. Further investigation of monocyte function, such as migration, cytokine secretion, and antiviral response in the context of dengue and obesity, will represent a significant contribution in the elucidation of the obesity–dengue association.

## Figures and Tables

**Table 1:** Anthropometric data of study’s participants.

	Lean		Obesity	
	DENV IgM Neg	DENV IgM Pos	DENV IgM Neg	DENV IgM Pos
Sex (F/M)	6/4 ^a^	3/2 ^a^	11/8 ^a^	8/7 ^a^
Age (years)	27.6 ± 6.2 ^a^	26.2 ± 5.5 ^a^	28.6 ± 7.1 ^a^	26.1 ± 4.7 ^a^
Body mass (kg)	62.6 ± 6.3 ^a^	60.5 ± 8.9 ^a^	94.9 ± 14.3 ^b^	91.8 ± 10.7 ^b^
Height (cm)	166.6 ± 4.7 ^a^	167.6 ± 10.2 ^a^	166.5 ± 8.5 ^a^	163.0 ± 7.3 ^a^
BMI (kg/m^2^)	22.5 ± 1.4 ^a^	21.5 ± 1.7 ^a^	34.1 ± 3.8 ^b^	34.5 ± 2.5 ^b^
Waist circumference (cm)	75.3 ± 5.9 ^a^	72.1 ± 4.4 ^a^	99.0 ± 9.3 ^b^	98.6 ± 9.9 ^b^
Body fat (%)	30.8 ± 8.5 ^a^	29.0 ± 8.1 ^a^	41.3 ± 6.3 ^b^	43.9 ± 7.0 ^b^
Fat mass (kg)	18.0 ± 8.5 ^a^	16.4 ± 4.3 ^a^	36.1 ± 6.2 ^b^	38.7 ± 7.0 ^b^
Visceral fat mass (g)	215.2 ± 177.3 ^a^	168.6 ± 115.2 ^a^	1146.5 ± 553.8 ^b^	1128.1 ± 565.0 ^b^
BFI (kg/m^2^)	6.6 ± 1.7 ^a^	5.9 ± 1.7 ^a^	12.7 ± 2.1 ^b^	14.7 ± 3.1 ^c^
Fat free mass (kg)	41.5 ± 8.5 ^a^	41.2 ± 9.5 ^a^	53.4 ± 10.3 ^b^	49.3 ± 8.2 ^b^

Data expressed as mean ± SD. *p* < 0.05 are represented by different superscript letters, two-way Anova, Šídák post-hoc. DENV = dengue virus, neg = negative, pos = positive, F = female, M = male, BMI = body mass index, BFI = body fat index.

**Table 2:** Endocrine and metabolic data of the study’s participants.

	Lean		Obesity	
	DENV IgM Neg	DENV IgM Pos	DENV IgM Neg	DENV IgM Pos
Fasting glucose (mg/dL)	81.9 ± 5.5 ^a^	83.7 ± 7.3 ^a^	84.8 ± 6.0 ^a^	87.2 ± 4.3 ^a^
Fasting insulin (µUI/mL)	5.9 ± 0.5 ^a^	4.9 ± 2.9 ^a^	14.9 ± 8.0 ^b^	18.1 ± 9.3 ^b^
HOMA- IR (mmol.µUI/L^2^)	1.2 ± 0.1 ^a^	1.0 ± 0.6 ^a^	3.1 ± 1.7 ^b^	3.9 ± 2.1 ^b^
HOMA- β (mmol.µUI/L^2^)	106.2 ± 16.7 ^a^	110.3 ± 93.7 ^a^	255.2 ± 128.8 ^b^	264.3 ± 112.2 ^b^
Cholesterol (cm)	172.3 ± 37.5 ^a^	161.3 ± 24.6 ^a^	188.5 ± 41.6 ^a^	162.6 ± 25.5 ^a^
HDL (mg/dL)	57.7 ± 6.2 ^a^	62.7 ± 17.9 ^a^	44.9 ± 12.8 ^b^	41.0 ± 14.8 ^b^
LDL (mg/dL)	95.9 ± 33.9 ^a^	76.4 ± 17.4 ^a^	112.6 ± 31.7 ^a^	109.3 ± 33.4 ^a^
VLDL (mg/dL)	18.6 ± 8.9 ^a,b^	22.1 ± 4.5 ^a,b^	31.0 ± 20.6 ^a^	17.1 ± 5.8 ^b^
Triglycerides (mg/dL)	78.5 ± 36.1 ^a^	93.3 ± 50.5 ^a,b^	144.6 ± 62.5 ^b^	113.4 ± 47.6 ^a,b^
Leptin (ng/mL)	2.4 ± 1.1 ^a^	2.7 ± 0.7 ^a,b^	3.3 ± 0.2 ^b^	3.0 ± 0.3 ^a,b^

Data expressed as mean ± SD. *p* < 0.05 are represented by different superscript letters, two-way Anova, Šídák post-hoc. DENV = dengue virus, neg = negative, pos = positive, HOMA-IR = homeostatic model assessment of insulin resistance, HOMA-β = homeostatic model assessment of β-cell function, HDL = high-density lipoprotein, LDL = low-density lipoprotein, VLDL = very low-density lipoprotein.

**Table 3:** Global and differential leukocyte count of the study’s participants.

	Lean		Obesity	
Cell Count (Cell/mm^3^)	DENV IgM Neg	DENV IgM Pos	DENV IgM Neg	DENV IgM Pos
Leukocytes	6.04 ± 1.46	6.37 ± 1.71	6.92 ± 1.55	6.50 ± 1.71
Neutrophils	2.99 ± 9.00	3.62 ± 1.13	3.59 ± 1.16	2.95 ± 0.82
Lymphocytes	2.43 ± 0.81	2.18 ± 0.70	2.67 ± 0.59	2.69 ± 0.74
Monocytes	0.37 ± 0.12	0.34 ± 0.03	0.38 ± 0.08	0.37 ± 0.11

Data expressed as mean ± SD. DENV = dengue virus, neg = negative, pos = positive.

## Data Availability

The data presented in this study are available on request from the corresponding author.
